# Bamboo Polyphenols Protect Against *Salmonella* Enteritidis in Chickens by Modulating Inflammation, Barrier Integrity, and Microbiota

**DOI:** 10.3390/ani16091290

**Published:** 2026-04-22

**Authors:** Qiaomei Liao, Long Zheng, Jinyang Huang, Bingjie Zou, Xidong He, Yijian Wu

**Affiliations:** 1 College of Animal Science, Fujian Agriculture and Forestry University, Fuzhou 350002, China; 52306023021@fafu.edu.cn (Q.L.); zl20011025bx@163.com (L.Z.); huangjinyang2001@163.com (J.H.); 2 Fujian Sunvet Bio-Technology Co., Ltd., Nanping 354100, China; zoubingjie6677@163.com; 3 Fujian Sunnzer Biotechnology Development Co., Ltd., Nanping 354100, China; 62506023013@fafu.edu.cn; 4 University Key Laboratory for Integrated Chinese Traditional and Western Veterinary Medicine and Animal Healthcare in Fujian Province, Fuzhou 350002, China; 5 Fujian Key Laboratory of Traditional Chinese Veterinary Medicine and Animal Health, Fujian Agriculture and Forestry University, Fuzhou 350002, China

**Keywords:** bamboo polyphenols, *Salmonella* Enteritidis, chicks, inflammation modulation, gut microbiota

## Abstract

*Salmonella* Enteritidis (SE) infections in poultry threaten animal health and food safety. Antibiotic resistance makes alternative treatments necessary. Bamboo polyphenols (BP), recovered from bamboo vinegar—a byproduct of bamboo carbonization—represent a sustainable and eco-friendly candidate for combating avian salmonellosis. We tested BP against SE using laboratory tests and a chick model. BP showed a minimum inhibitory concentration of 1:256 against SE. We infected chicks with 1.8 × 10^8^ CFU per bird. The results indicated that adding 0.2% (*v*/*v*) BP to drinking water demonstrated optimal efficacy for prevention. Adding 0.4% (*v*/*v*) BP demonstrated optimal efficacy for treatment. Prophylactic BP administration effectively prevented SE-induced mortality and tissue damage. As a therapeutic agent, BP performed comparably to berberine. BP lowered the bacterial load in organs and increased chick survival to 96%. At the transcriptional level, BP administration downregulated the TLR4/MyD88/NF-κB pathway. It also improved antioxidant levels, strengthened the intestinal barrier, and restored healthy gut bacteria. These results indicate that BP could serve as a potential and sustainable feed additive to reduce SE infection in poultry.

## Simple Summary

*Salmonella* infections in chickens cause large economic losses in the poultry industry and threaten food safety. While antibiotics are commonly used to control these infections, their overuse leads to the rise of drug-resistant bacteria. This study explores bamboo polyphenols, a natural substance recovered from bamboo charcoal waste, as a sustainable and eco-friendly alternative. Our results show that adding bamboo polyphenols to drinking water effectively protects chicks from *Salmonella* by killing the bacteria and reducing gut inflammation. Bamboo polyphenols also strengthen the intestinal barrier and improve the balance of healthy gut bacteria. This research provides a green strategy to protect poultry health and reduce the need for antibiotics.

## 1. Introduction

*Salmonella* Enteritidis (SE) is a globally prevalent zoonotic pathogen that inflicts severe economic losses on the poultry industry by causing acute enteritis and systemic sepsis in young chicks. Additionally, SE can contaminate poultry products and enter the human food chain, posing a persistent threat to public health [1,2]. Upon colonizing the avian intestinal tract, SE disrupts tight junctions, elevates mucosal permeability, and induces microbial dysbiosis. These alterations exacerbate intestinal damage and facilitate the systemic dissemination of the pathogen.

Traditional management of avian salmonellosis relied heavily on antibiotics. Yet, chronic overuse has driven the rise of multidrug-resistant SE strains. It also causes undesirable drug residues in animal products [3,4,5]. These challenges not only jeopardize food safety but also compromise clinical treatment efficacy, highlighting the urgent need for safe and eco-friendly alternatives.

Natural plant-derived bioactive compounds, particularly phenolic compounds, offer broad-spectrum efficacy and low residual risks [6,7,8,9,10]. These compounds serve as sustainable alternatives to combat *Salmonella* infections in poultry without inducing antimicrobial resistance [11]. Bamboo polyphenols (BP) are value-added bioactive compounds extracted from the low-boiling fraction of bamboo vinegar, which is a major liquid byproduct generated during the pyrolysis and carbonization of bamboo. Utilizing BP not only provides a natural antimicrobial source but also promotes the recycling of agricultural and forestry waste, aligning with sustainable production practices. Bamboo polyphenols (BP) contain a complex mixture of active molecules. Our BP extract primarily consists of natural organic acids and plant polyphenols. Acetic acid makes up more than 16% of the extract. Guaiacol and 4-methylguaiacol are the main polyphenols. The extract also contains various ketones. These molecules work together to damage bacterial cell membranes. They stop bacteria from multiplying. Using bamboo waste to make these additives creates a circular economy. It reduces environmental pollution from forestry waste. Past studies show BP has strong anti-inflammatory and antioxidant properties, alongside direct in vitro antibacterial activity against enteric pathogens like *Salmonella* and *Escherichia coli* [12,13,14,15,16]. These multifaceted bioactivities suggest that BP may alleviate intestinal disorders by simultaneously inhibiting pathogen proliferation and modulating host immunity [17].

However, researchers have not evaluated the in vivo protective effects of BP against avian salmonellosis. Its specific regulatory impact on intestinal barrier integrity and gut microbiota homeostasis remains unclear. While previous nutritional interventions have shown success in modulating these intestinal factors [18,19], whether water-soluble BP from bamboo vinegar can offer similar protective associations requires investigation.

This study aimed to (1) establish a standardized SE infection model in broiler chicks [20]; (2) determine the optimal preventive and therapeutic concentrations of BP delivered via drinking water; (3) evaluate the relative efficacy of BP alongside a clinical antibiotic (enrofloxacin) [21] and a classical plant-derived agent (berberine); and (4) explore the potential associations between BP administration and inflammatory signaling, oxidative stress, barrier function, and microbiome modulation.

## 2. Materials and Methods

### 2.1. Animal Ethics and Husbandry

All experimental procedures received approval from the Academic Committee of Fujian Agriculture and Forestry University (Approval No. PZCASFAFU24011). We strictly followed the national guidelines for laboratory animal care in China. One-day-old healthy white-feather broiler chicks (Sunner Development Co., Ltd., Nanping, China) underwent a 3-day acclimation period. The chicks did not receive any vaccines prior to the experiment. They were housed in a controlled environment (32–34 °C, 55 ± 5% relative humidity) with free access to sterile water and an antibiotic-free basal diet.

### 2.2. Bacterial Strains and BP Preparation

The SE strain (designated as P165-1) used in this study was originally isolated from a cloacal swab. This sample was collected at a maternal rearing farm (12W3) belonging to the Fujian Sunner Group (Nanping, China). The strain was resuscitated and purified on MacConkey and SS agar plates. A standard curve correlating the OD_600_ values of the SE suspension with the viable bacterial concentration (CFU/mL) was constructed. The minimum inhibitory concentration (MIC) and minimum bactericidal concentration (MBC) of BP (Fujian Sunvet Bio-Technology Co., Ltd., Nanping, China), which was provided as a refined liquid stock solution, were determined using a microbroth dilution method. Serial dilutions of the BP stock solution were prepared in sterile Mueller–Hinton (MH) broth (Hopebio, Qingdao, China) and inoculated with SE (5 × 10^5^ CFU/mL), with a kanamycin sulfate solution (Hopebio, Qingdao, China) serving as the positive control. The preparation of BP involved a standardized industrial process. First, bamboo underwent pyrolysis to produce raw bamboo vinegar. Subsequent distillation of this raw vinegar yielded the BP extract. The final BP stock solution contained ≥10% plant polyphenols. Guaiacol (≥3.8%) and 4-methylguaiacol (≥3.2%) were the primary phenolic compounds. The solution also contained ≥20% natural organic acids, with acetic acid accounting for ≥16%. The remaining components consisted of ketones, furan derivatives, and water.

### 2.3. Experimental Design and Animal Trials

#### 2.3.1. Model Establishment

Four SE challenge doses were evaluated: 1.8 × 10^6^ (G1), 1.8 × 10^7^ (G2), 1.8 × 10^8^ (G3), and 1.8 × 10^9^ (G4) CFU per chick. A total of 125 four-day-old chicks were randomly allocated into a blank control group (PBS) and four challenge groups (*n* = 25 per group). Chicks in the challenge groups were orally gavaged with their respective SE doses once daily on days 5, 6, and 7 (3 consecutive days). Clinical symptoms (scored twice daily according to the criteria in Table 1), mortality, body weight, and organ bacterial loads were monitored for 14 days to determine the optimal challenge dose for subsequent experiments.

#### 2.3.2. Dose Screening

The experiment randomized 300 one-day-old chicks into eight groups. Seven treatment and challenge groups contained 40 chicks each. Within these seven groups, 20 chicks were utilized for clinical observation and 20 for sampling. The blank control group contained only 20 chicks for sampling. This blank control group omitted the clinical observation subset to minimize animal usage, because healthy birds exhibit no clinical symptoms. The groups included a blank control, a positive control (SE-challenged, untreated), three preventive BP groups (0.1%, 0.2%, and 0.4%, *v*/*v*, of the BP stock solution in drinking water), and three therapeutic BP groups (0.2%, 0.4%, and 0.8%, *v*/*v*, of the BP stock solution in drinking water). Preventive BP was administered on days 2–5, and therapeutic BP was administered on days 9–11. All challenged groups received SE via oral gavage (1.8 × 10^8^ CFU/chick/day) on days 6–8. The earlier model establishment phase infected chicks on days 5–7 to confirm early-age susceptibility. However, the dose screening and efficacy trials required a prophylactic treatment phase. This preventive phase occupied days 2–5 to ensure sufficient accumulation of BP in the intestinal tract. Therefore, the infection window for the later trials shifted to days 6–8. This slight adjustment accommodated the preventive study design without altering the acute infection model.

#### 2.3.3. Efficacy Trial

For the formal trial, 280 healthy chicks were divided into seven groups (*n* = 40/group, allocating 20 for clinical observation and 20 for sampling): a blank control (BC), a BP control (BPC, 0.4% *v*/*v* BP), a positive control (PC), a BP preventive group (BP-P, 0.2% *v*/*v* BP), a BP therapeutic group (BP-T, 0.4% *v*/*v* BP), a berberine hydrochloride treatment group (BH-T, 0.2 g/L; Jiupeng Pharmaceutical Co., Ltd., Handan, China), and an enrofloxacin hydrochloride treatment group (EH-T, 0.1 g/L; Jiupeng Pharmaceutical Co., Ltd., Handan, China). All challenged groups were gavaged with SE (1.8 × 10^8^ CFU/chick/day) on days 6–8.

### 2.4. Sample Collection

Sampling protocols differed across the three experimental phases. During the model establishment trial (Section 2.3.1), chicks were randomly selected for necropsy on days 1, 3, 7, and 14 post-challenge to evaluate gross pathological lesions and organ bacterial loads. During the BP dose-screening trial (Section 2.3.2), chicks were euthanized on days 1, 3, and 7 post-treatment to assess pathological damage, bacterial loads, inflammatory cytokines, and oxidative stress. During the formal efficacy trial (Section 2.3.3), six chicks per group were randomly selected and euthanized on days 1, 3, and 7 post-treatment. Blood was collected, and serum was separated by centrifugation (3000 rpm, 15 min, 4 °C; Eppendorf 5810R, Eppendorf, Hamburg, Germany) and stored at −80 °C. Liver, ileum, and cecal tissues were harvested. Portions were fixed in 4% paraformaldehyde for histopathology, while the remainder and cecal contents were snap-frozen in liquid nitrogen and stored at −80 °C. For bacterial load quantification, 0.1 g of cecal contents was homogenized in 1 mL of PBS, serially diluted, and spread onto SS agar plates to determine log_10_ CFU/g. The spleen index was calculated as (spleen weight/body weight) × 100%.

### 2.5. Analytical Methods

#### 2.5.1. Histopathological Analysis

Fixed tissues were dehydrated (KH-TS, Hubei Xiaogan Kuohai Medical Technology Co., Ltd., Xiaogan, China), embedded in paraffin (KH-BL), sectioned at 5 μm (HM 325, Epredia, Kalamazoo, MI, USA), and stained with hematoxylin and eosin (Solarbio, Beijing, China). Intestinal morphology was measured using ImageJ software (version 1.x, National Institutes of Health, Bethesda, MD, USA).

#### 2.5.2. Gene Expression

Total RNA was extracted from tissue samples using the RNA Isolation Kit V2 (Vazyme Biotech Co., Ltd., Nanjing, China). RNA purity and concentration were verified before reverse transcription. qRT-PCR was performed on a CFX96 thermal cycler (Bio-Rad, Hercules, CA, USA) to measure the mRNA expression of *TNF-α*, *IL-1β*, *IL-6*, *IL-10*, *TLR4*, *MyD88*, and *NF-κB*. Primer sequences are provided in Table 2, with *β-actin* as the reference gene.

#### 2.5.3. Biochemical and ELISA Assays

Serum T-AOC, SOD, GSH-Px, MDA, and LDH were measured using commercial kits (Nanjing Jiancheng Bioengineering Institute, Nanjing, China) and read on a microplate reader (Tecan Infinite M200, Tecan, Männedorf, Switzerland). Cecal ZO-1, Occludin, and sIgA levels were quantified using ELISA kits (Shanghai Enzyme-Linked Immunobiotechnology Co., Ltd., Shanghai, China) following centrifugation (12,000 rpm, 15 min, 4 °C).

#### 2.5.4. 16S rRNA Sequencing

Total genomic DNA was extracted from cecal contents. The V3–V4 region was amplified (primers 341F/806R), and amplicons were sequenced on the Illumina NovaSeq PE250 platform (Illumina, San Diego, CA, USA). Data were analyzed using the Python 3 (https://www.python.org/) implementation of DADA2 (https://benjjneb.github.io/dada2/, accessed on 12 April 2026), and LEfSe (version 1.1.2) was applied to identify differentially abundant taxa.

### 2.6. Statistical Analysis

All statistical analyses were performed using SPSS 26.0 software (IBM Corp., Armonk, NY, USA). One-way ANOVA followed by Duncan’s multiple range test was used for pairwise comparisons. A *p*-value < 0.05 was considered statistically significant. Graphs were generated using GraphPad Prism 10.5 software (GraphPad Software, San Diego, CA, USA).

## 3. Results

### 3.1. In Vitro Antimicrobial Activity and Establishment of the SE Infection Model

**Figure 1:**
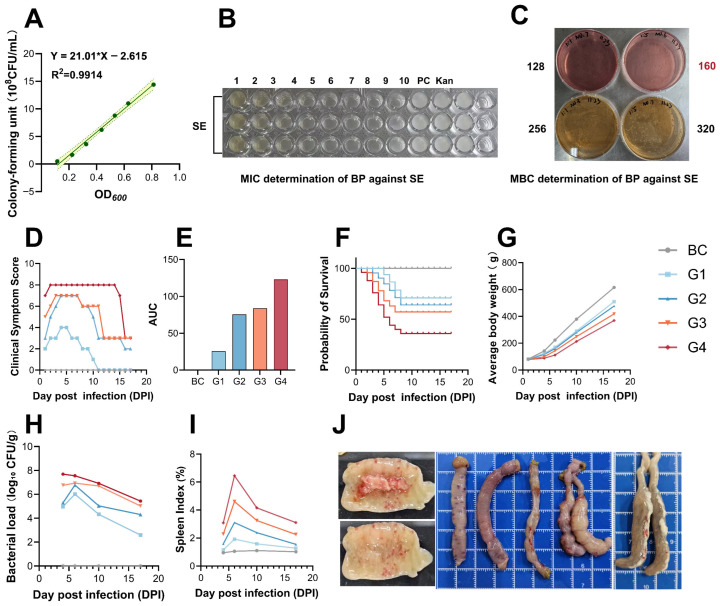
Establishment of the bacterial quantification method, antimicrobial activity of BP, and dose-dependent effects of SE challenge on growth, immunity, and bacterial load in chicks: (**A**) Standard curve between OD_600_ value and viable SE concentration. (**B**) Minimum inhibitory concentration (MIC) determination of BP against SE. (**C**) Minimum bactericidal concentration (MBC) determination of BP against SE. (**D**–**G**) Dynamic changes in clinical symptom scores (**D**), AUC of clinical symptom scores (AUC: area under the curve), (**E**) survival probability (**F**), and average body weight (**G**) of chicks challenged with different SE doses (G1–G4) vs. BC. (**H**) Temporal variation in cecal bacterial load (log_10_ CFU/g) in SE-challenged chicks. (**I**) Spleen index changes in SE-challenged chicks. (**J**) Morphological characteristics of cecal lesions (caseous plug formation) induced by the optimal SE challenge dose (1.8 × 10^8^ CFU/chick). BC: blank control; G1–G4: different concentration challenge groups. Each blue grid square in panel (**J**) represents 1 cm.

In vitro antimicrobial activity. A strong linear correlation was observed between the OD_600_ value and viable SE concentration (*R*^2^ = 0.9914). Based on this curve, preparing the 1.8 × 10^8^ CFU/mL inoculum required a target OD_600_ value of approximately 0.21. The in vitro MIC and MBC of the BP stock solution against SE were identified as 1:256 and 1:160 dilutions, respectively (Figure 1B,C). Based on the stock solution composition, the 1:256 dilution corresponds to an effective polyphenol concentration of approximately 390 μg/mL.

**Figure 2:**
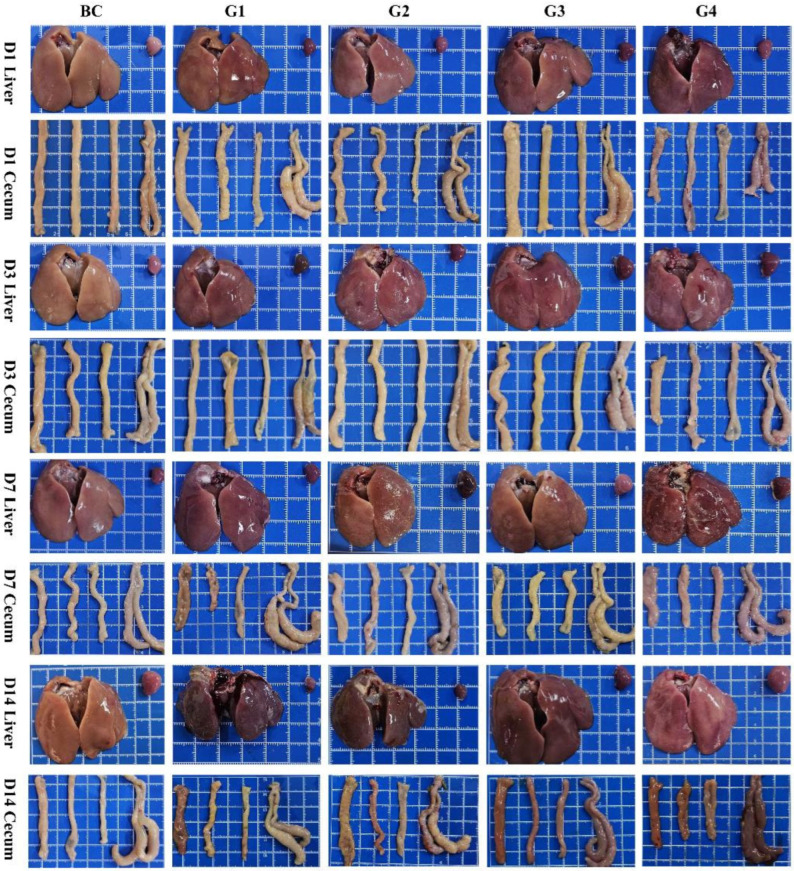
Typical macroscopic pathological changes in the liver and cecum in chicks challenged with different doses of SE (G1–G4) compared to the blank control (BC) at days 1, 3, 7, and 14 post-infection. Each blue grid square represents 1 cm.

In vivo model establishment. In vivo, increasing SE challenge doses linearly exacerbated clinical symptoms, reduced survival rates, and increased both spleen indices and cecal bacterial loads (Figure 1D–I). Notably, the 1.8 × 10^8^ CFU/chick dose induced consistent, typical SE-associated pathological lesions. Macroscopic observation revealed progressive liver hepatomegaly, severe congestion, and the formation of characteristic cecal “caseous plugs” over the 14-day post-infection period (Figure 1J and Figure 2) [22,23]. This 1.8 × 10^8^ CFU dose caused moderate mortality. The higher dose (G4) caused excessive acute mortality. The lower doses (G1 and G2) failed to produce stable clinical symptoms. Therefore, the 1.8 × 10^8^ CFU dose was selected as the optimal challenge dose for all subsequent efficacy trials.

### 3.2. Optimization of Preventive and Therapeutic BP Concentrations

**Figure 3:**
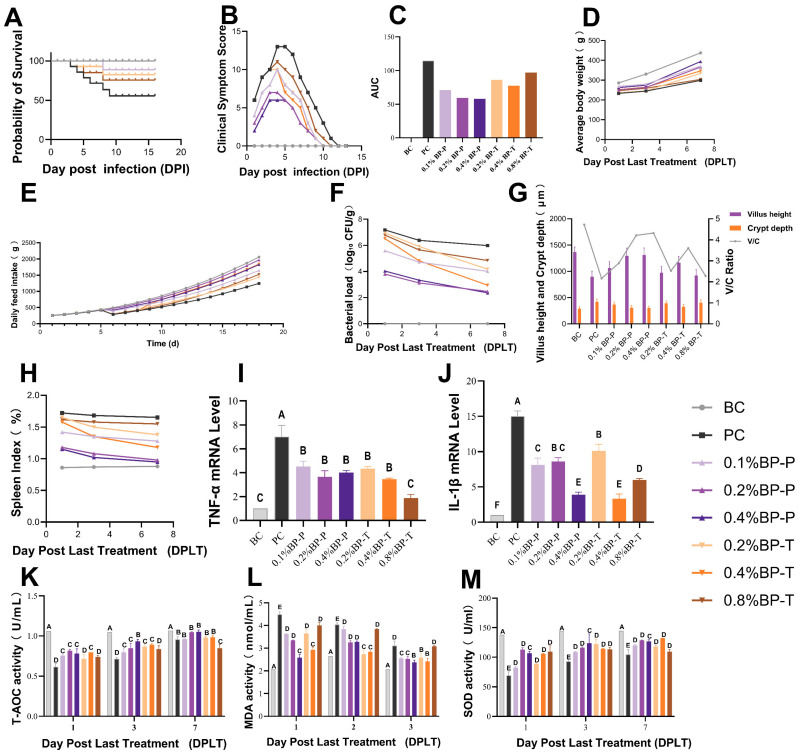
Effects of graded concentrations of drinking water-administered BP on SE-infected broiler chicks: (**A**) Survival rate of chicks post-infection. (**B**) Clinical severity scores of SE-infected chicks over time. (**C**) AUC: area under the curve. (**D**) Average body weight. (**E**) Daily feed intake of chicks. (**F**) Organ bacterial load (log_10_ CFU/g tissue) at the end of the post-treatment observation period. (**G**) Ileal villus height, crypt depth, and villus height/crypt depth (V/C) ratio. (**H**) Spleen Index (%). (**I**,**J**) mRNA expression levels of pro-inflammatory cytokines *TNF-α* and *IL-1β* in cecal tissue. (**K**–**M**) Serum antioxidant parameters: T-AOC, MDA content, and SOD activity. PC, positive control; BP-P, BP preventive administration; BP-T, BP therapeutic administration. Values with different superscript letters within the same index indicate significant differences (*p* < 0.05) by Duncan’s multiple range test. Values with different superscript letters indicate significant differences (*p* < 0.05) by Duncan’s multiple range test. Data are presented as the mean ± SD.

**Figure 4:**
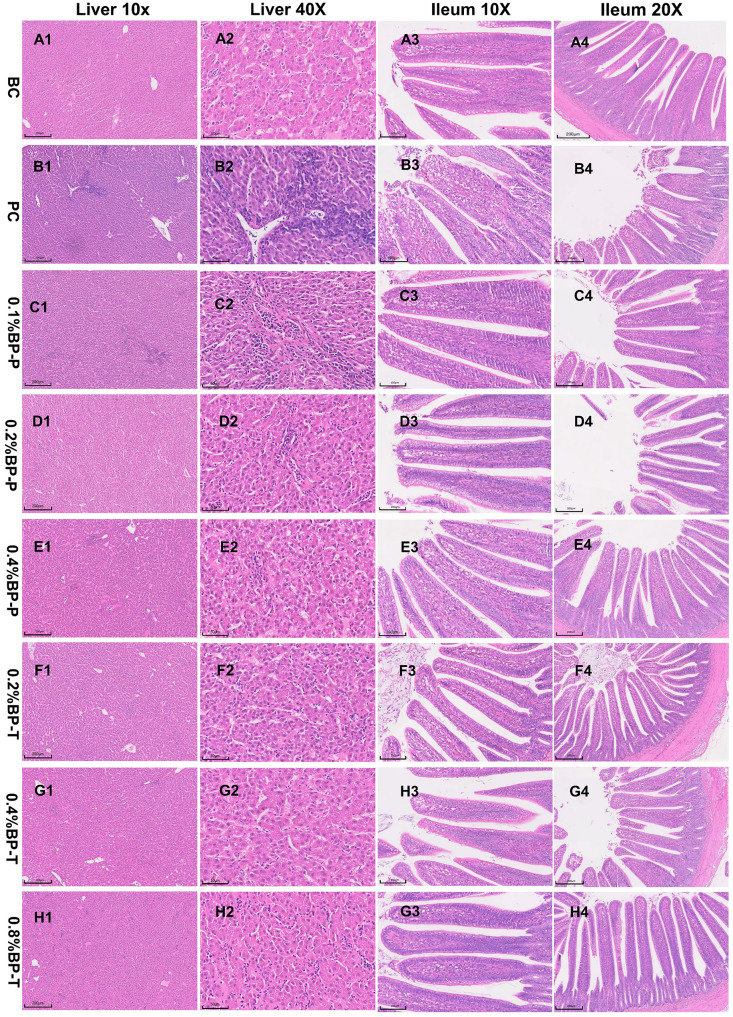
Histopathological changes in liver and ileum in SE-infected broiler chicks treated with graded concentrations of drinking water-administered BP (**A1–4**–**H1–4**). Representative hematoxylin and eosin (H&E)-stained sections of liver (scale bar: 200 μm and 50 μm) and ileum (scale bar: 200 μm and 100 μm).

In the preventive model, both 0.2% and 0.4% BP ensured a 100% survival rate, reduced organ bacterial load, and restored intestinal morphological structure. Furthermore, histopathological evaluation confirmed that these BP interventions noticeably attenuated hepatic necrosis and ileal mucosal inflammation (Figure 3 and Figure 4) compared with the untreated positive control (PC) group. Additionally, BP supplementation at 0.2% and 0.4% successfully attenuated the overexpression of pro-inflammatory cytokines in cecal tissues and ameliorated serum oxidative stress indicators. In the therapeutic model, 0.4% BP yielded the highest survival rate (96%) and optimal physiological recovery. Higher concentrations (0.8%) proved less effective. Consequently, considering both efficacy and economic feasibility, 0.2% and 0.4% BP were established as the optimal preventive and therapeutic concentrations, respectively.

### 3.3. Efficacy of BP Against SE Infection

**Figure 5:**
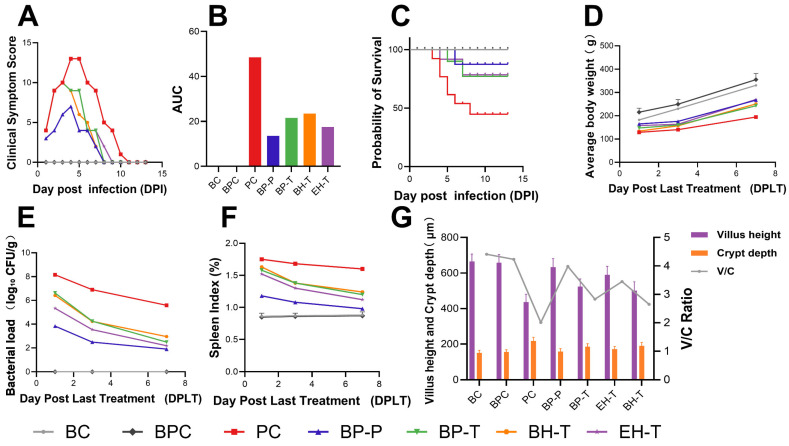
Efficacy of different treatments against SE infection in chicks: (**A**) Clinical symptom scores of chicks in each group. (**B**) AUC: area under the curve. (**C**) Cumulative survival rate of chicks in each group. (**D**) Daily body weight. (**E**) Dynamic changes in the organ bacterial load of SE-infected chicks in each group. (**F**) Spleen Index (%). (**G**) Effects of different treatments on ileal mucosal morphology (villus height and crypt depth) of SE-infected chicks. BC = blank control; PC = positive control; BP-P = 0.2% BP prophylactic; BP-T = 0.4% BP therapeutic; BH-T = berberine hydrochloride therapeutic; EH-T = enrofloxacin hydrochloride therapeutic.

**Figure 6:**
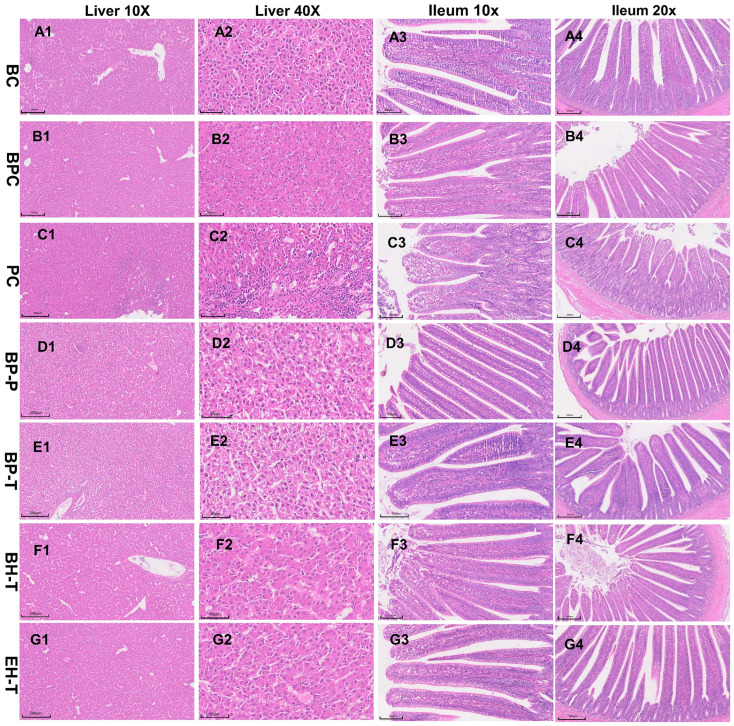
Histopathological changes in the liver and ileum of SE-infected broiler chickens after treatment with various drugs (**A1–4**–**G1–4**). Representative hematoxylin and eosin (H&E)-stained sections of liver (scale bar: 200 μm and 50 μm) and ileum (scale bar: 200 μm and 100 μm).

SE challenge in the PC group caused severe clinical symptoms, decreased average daily body weight, and disrupted intestinal morphology (Figure 5). Treatment with BP-P and BP-T significantly reversed these detriments. The BP-P regimen provided complete protection against mortality, effectively preserving intestinal structure. Therapeutically, BP-T performed similarly to the natural extract BH-T. Histopathological analysis confirmed that BP interventions noticeably reduced hepatic necrosis and ileal mucosal inflammation (Figure 6).

### 3.4. Modulation of Inflammation and Oxidative Stress

**Figure 7:**
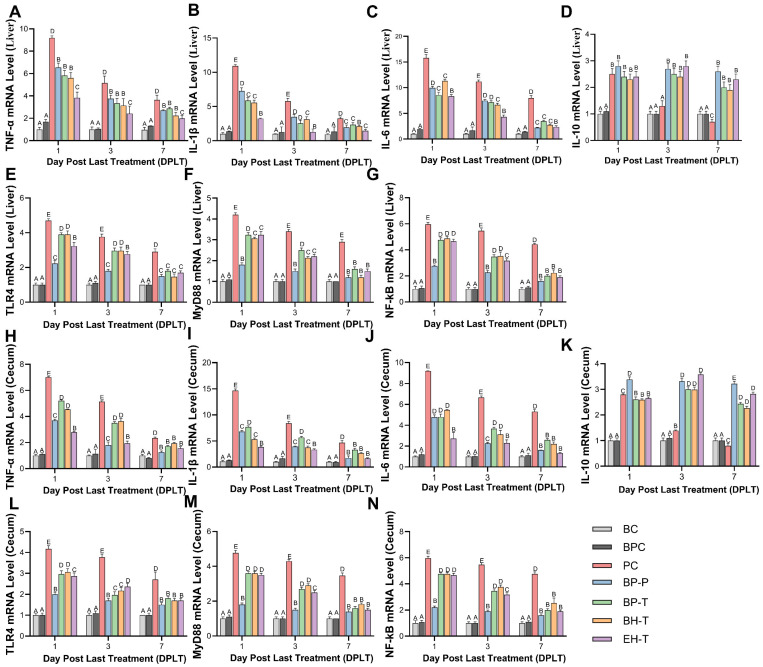
Expression levels of inflammatory factors (**A**–**D**,**H**–**K**) and NF-κB signaling pathway molecules (**E**–**G**,**L**–**N**) in liver and cecum tissues of chicks in each group: (**A**–**G**) liver tissue; (**H**–**N**) cecum tissue. Values with different superscript letters indicate significant differences (*p* < 0.05) by Duncan’s multiple range test. Data are presented as the mean ± SD.

**Figure 8:**
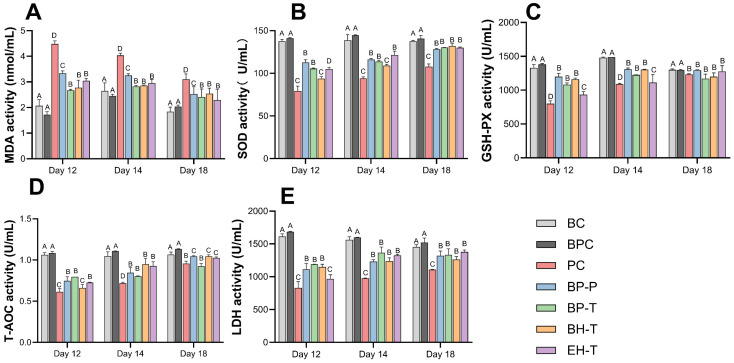
Serum oxidative stress indicators of chicks in each group: (**A**) T-AOC activity; (**B**) SOD activity; (**C**) GSH-Px activity; (**D**) MDA content; (**E**) LDH activity. Values with different superscript letters indicate significant differences (*p* < 0.05) by Duncan’s multiple range test. Data are presented as the mean ± SD.

SE infection upregulated pro-inflammatory cytokines (*TNF-α*, *IL-1β*, and *IL-6*) and *TLR4*/*MyD88*/*NF-κB* pathway genes while suppressing the anti-inflammatory cytokine IL-10 (Figure 7). Both BP-P and BP-T significantly mitigated this overactivation, performing comparably to EH-T and surpassing BH-T. Furthermore, BP administration restored serum antioxidant enzyme activities (SOD, GSH-Px) and reduced cellular damage markers (MDA, LDH), effectively neutralizing SE-induced oxidative stress (Figure 8).

### 3.5. Fortification of the Intestinal Barrier and Microbiota

**Figure 9:**
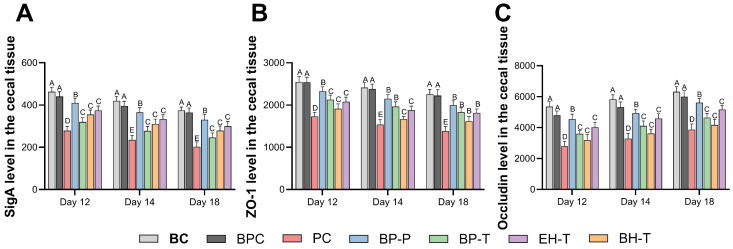
Expression levels of intestinal barrier function-related proteins in the cecal tissue of chicks in each group: (**A**) sIgA levels; (**B**) ZO-1 levels; (**C**) Occludin levels. Values with different superscript letters indicate significant differences (*p* < 0.05) by Duncan’s multiple range test. Data are presented as the mean ± SD.

**Figure 10:**
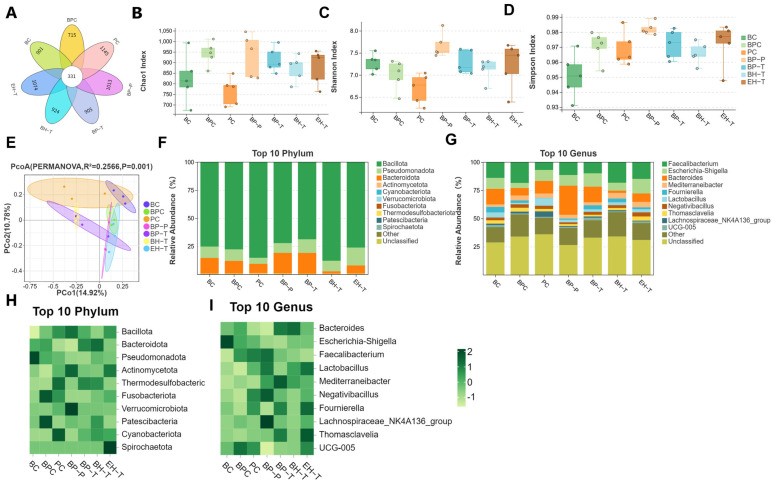
Cecal microbiota composition and diversity in chicks: (**A**) Venn diagram illustrating the number of operational taxonomic units (OTUs). (**B**–**D**) Alpha diversity indices: Chao1 (**B**), Shannon (**C**), and Simpson (**D**). (**E**) Beta diversity analysis based on OTUs using principal coordinate analysis (PCoA). (**F**,**G**) Stacked bar charts and (**H**,**I**) heatmaps showing the relative abundance of microbiota at the phylum (**F**,**H**) and genus (**G**,**I**) levels. Values are presented as the mean ± SEM (*n* = 5).

**Figure 11:**
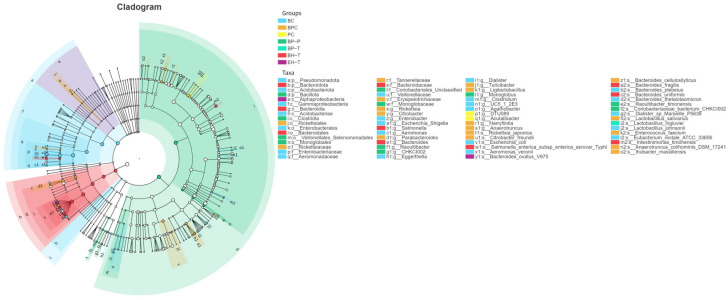
LEfSe cladogram of cecal microbiota. The cladogram illustrates the phylogenetic distribution of significantly enriched microbial taxa.

**Figure 12:**
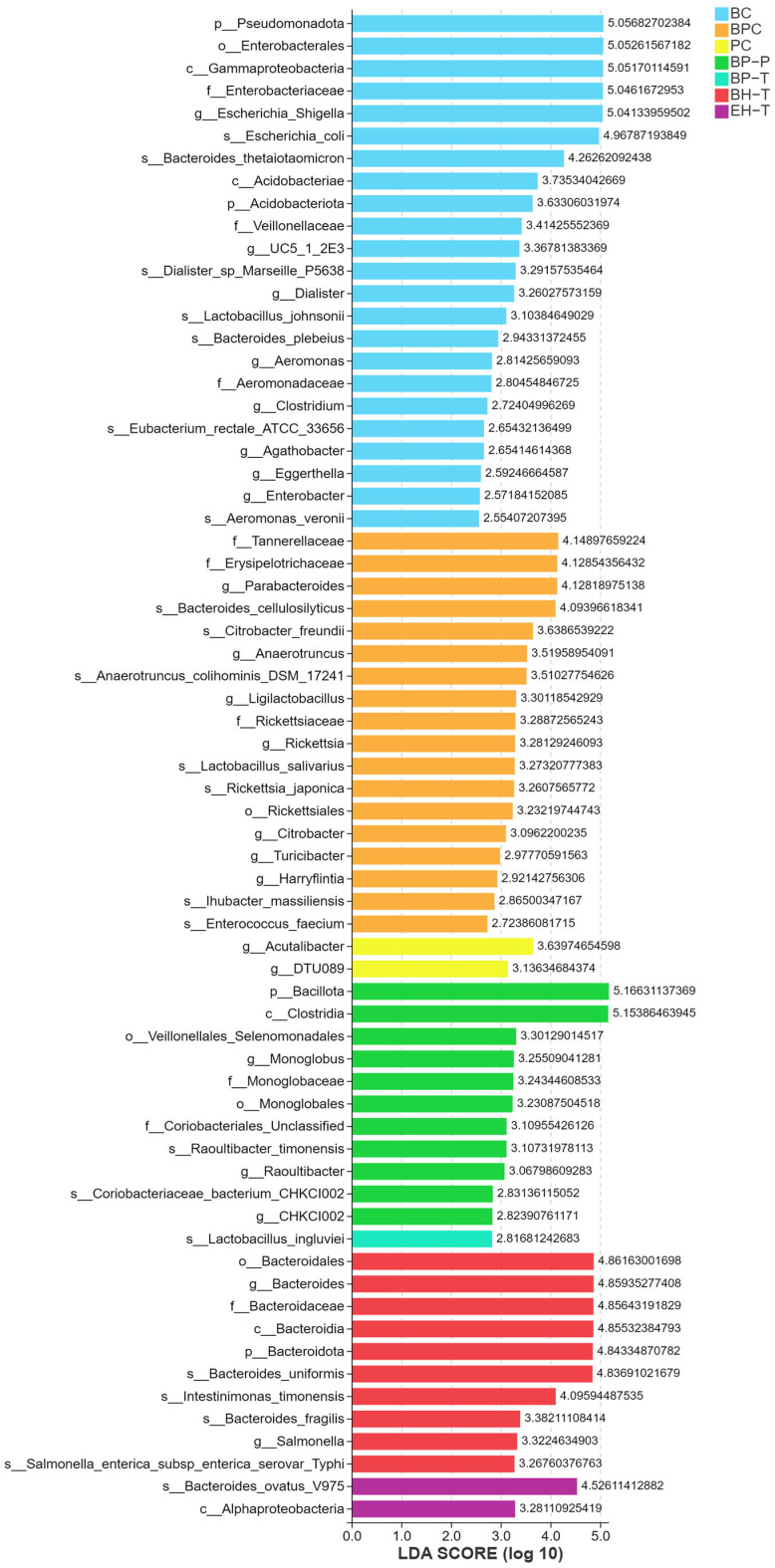
LEfSe bar chart of cecal microbiota. The chart shows the LDA scores of differentially abundant taxa (LDA > 2.0, *p* < 0.05).

SE severely depleted cecal tight junction proteins (ZO-1, Occludin) and sIgA. BP treatments significantly upregulated these markers, reinforcing the mucosal barrier (Figure 9). Microbiome sequencing revealed that SE exposure increased the abundance of Proteobacteria and pathogenic *Escherichia–Shigella* (Figure 10). BP interventions reversed this dysbiosis, significantly enriching beneficial taxa such as *Faecalibacterium* and *Lactobacillus* while repressing opportunistic pathogens, shifting the microbial community structure back toward that of healthy controls (Figure 11 and Figure 12).

## 4. Discussion

The persistent threat of SE in commercial poultry operations highlights the demand for effective non-antibiotic interventions [24,25]. Furthermore, the poultry industry is increasingly seeking feed additives derived from sustainable and circular economic models. Our study evaluated BP—a refined extract from bamboo carbonization waste—against SE infection in broiler chicks. We observed that BP delivers protection associated with direct antimicrobial action, immune modulation, barrier fortification, and microbiota restoration.

Establishing a reproducible animal infection model is critical for evaluating anti-infective agents. In our study, chicks infected with the optimal dose of 1.8 × 10^8^ CFU exhibited typical clinical symptoms and pathological changes consistent with previous reports [26], including mental depression, diarrhea, and anal contamination, alongside characteristic liver necrosis and cecal caseous plugs. This stable colonization validated our model for subsequent mechanistic investigations.

Optimizing the application dose is critical for maximizing the efficacy of bioactive compounds [27,28,29]. Our findings indicate a clear biphasic dose-response relationship in both preventing and treating SE infection, which aligns with observations from other phytogenic blend studies [30]. A 0.2% BP concentration proved economically optimal for disease prevention, while 0.4% was most effective for post-infection therapy. Higher concentrations produced weaker results. This is likely due to reduced water palatability and potential mucosal irritation. This phenomenon matches the hormetic effects frequently observed in plant extracts [31,32].

In our trials, prophylactic BP administration effectively prevented severe infection and mortality. This preventive capacity likely stems from BP’s ability to optimize gut health prior to pathogen exposure [33,34]. Enrofloxacin showed strong direct bactericidal activity post-infection [35,36]. However, comparing a preventive natural extract directly to a therapeutic antibiotic requires caution. They have different intervention windows. However, BP’s therapeutic efficacy rivaled that of berberine, demonstrating its value as a promising alternative that avoids antimicrobial resistance.

At the transcriptional level, the protective effect of BP was accompanied by the downregulation of the TLR4-driven MyD88/NF-κB inflammatory cascade. This pathway modulation reduces the hypersecretion of pro-inflammatory cytokines, protecting tissues from inflammatory damage [37,38,39]. The mechanism involves a direct biochemical interaction. Organic acids in BP lower the gut pH. This low pH strictly limits *Salmonella* colonization and growth in the ceca, an effect consistent with recent meta-analytical findings in broiler production [40]. Consequently, this bacterial inhibition reduces the release of lipopolysaccharide (LPS) into the gut environment. Less LPS means less activation of the TLR4 receptor. Furthermore, plant polyphenols penetrate host cells and inhibit kinase activity. This prevents the NF-κB complex from entering the cell nucleus. As a result, the production of inflammatory cytokines stops. BP also mitigates SE-induced oxidative stress by elevating endogenous antioxidant enzymes. This aligns with the known free-radical scavenging properties of plant polyphenols [41,42]. Recent nutrigenomic studies further confirm that dietary polyphenols alleviate physiological stress in broilers by concurrently regulating the antioxidant system and the NF-κB inflammatory cascade [43]. Additionally, BP administration was linked to higher levels of ZO-1, Occludin, and sIgA, effectively preventing bacterial translocation [44,45,46].

The gut microbiome was also reshaped following BP supplementation. BP promoted the proliferation of beneficial *Lactobacillus* and *Bifidobacterium* species while suppressing opportunistic enterobacteria, which promotes intestinal health and enhances host immunity [47]. These findings enrich the theoretical foundation for applying plant polyphenols to control avian salmonellosis [48,49,50,51].

Despite the positive outcomes, this study has limitations. First, our mechanistic data rely primarily on transcriptional changes and physiological markers. We have not yet identified the specific bioactive monomers in BP that drive the anti-SE effects. Future research should isolate and structurally characterize these components. Researchers also need to use protein-level validation and specific pathway inhibitors to confirm direct causal mechanisms. Second, deeper multi-omics analyses, such as metabolomics, will help clarify the complex interactions between BP, gut microbiota, and host immunity. Furthermore, these laboratory results need validation in large-scale field trials. Such trials will assess the long-term impacts of BP on poultry growth performance. The application method of BP also offers practical advantages. We administered BP through drinking water. Sick birds often reduce their feed intake, but they usually continue to drink water. Therefore, water administration ensures that infected chicks receive the treatment quickly. Moreover, farmers can easily apply this method in large-scale commercial poultry houses. It requires less labor than mixing solid additives into dry feed. Finally, exploring the synergistic effects of BP with other feed additives, like probiotics, is an important direction for future research.

## 5. Conclusions

Supplementation with the BP stock solution via drinking water—specifically 0.2% (*v*/*v*) for prevention and 0.4% (*v*/*v*) for treatment—effectively reduces the severity of SE infection in chicks. Derived from sustainable bamboo forestry waste, BP represents a promising, eco-friendly feed additive. Its benefits are closely associated with antibacterial activity, the dampening of host inflammation and oxidative stress, and the maintenance of barrier integrity and a balanced gut microbiome.

## Figures and Tables

**Table 1:** Scoring criteria for clinical symptoms of SE infection in chicks.

Clinical Parameter	Score 0 (Normal)	Score 1 (Mild)	Score 2 (Moderate)	Score 3 (Severe)
Mental status	Active and responsive	Decreased activity, lethargy	Obvious drowsiness, neck retraction	Inability to stand, moribund/coma
Diarrhea	Formed feces, normal color	Soft feces, minimal mucus	Watery feces, obvious mucus	Bloody or purulent watery feces
Anal contamination	Clean, no adhesion	Mild fecal adhesion	Moderate fecal contamination	Severe contamination (feather clumping)
Respiratory signs	Normal breathing, no rales	Mild tachypnea or occasional rales	Obvious dyspnea, open-mouth breathing	-
Feather appearance	Neat and glossy	Fluffy, slightly soiled	Severely unkempt, heavily soiled	-

**Table 2:** Primer sequences used for quantitative real-time PCR.

Target Gene	Primer Sequence (5′→3′)	Product Size (bp)
*IL-6*	F: TTCACCGTGTGCGAGAACAGC	80 bp
	R: CAGCCGTCCTCCTCCGTCAC	
*TNF-α*	F: TGATCGTGACACGTCTCTGC	55 bp
	R: CAACCAGCTATGCACCCCAG	
*IL1β*	F: AGCAGCCTCAGCGAAGAGACC	91 bp
	R: GTCCACTGTGGTGTGCTCAGAATC	
*IL10*	F: CGCTGTCACCGCTTCTTCA	90 bp
	R: TCCCGTTCTCATCCATCTTCTC	
*TLR4*	F: ACGGAAGGCTTTGGTTGGGATT	184 bp
	R: GATGTTGCTATCTGGTGCTTGGAA	
*MyD88*	F: TCTGGTGACTGTGGAGCAAGGAA	206 bp
	R: CCGCTTGTAGGAAGGCACTAATGG	
*NF-κB*	F: TCATCCACCGCCGCCACATT	232 bp
	R: GGCTGAGGAAGGCACTGAAGTC	
*β-actin*	F: GAGAAATTGTGCGTGACATCA	107 bp
	R: CCTGAACCTCTCATTGCCA	

Note: F, forward; R, reverse.

## Data Availability

The data that support the findings of this study are available from the corresponding author upon reasonable request.
